# Transcutaneous auricular vagus nerve immediate stimulation treatment for treatment-resistant depression: A functional magnetic resonance imaging study

**DOI:** 10.3389/fneur.2022.931838

**Published:** 2022-09-01

**Authors:** Yue Ma, Zhi Wang, Jiakai He, Jifei Sun, Chunlei Guo, Zhongming Du, Limei Chen, Yi Luo, Deqiang Gao, Yang Hong, Lei Zhang, Yong Liu, Jiliang Fang

**Affiliations:** ^1^Guang'anmen Hospital, China Academy of Chinese Medical Sciences, Beijing, China; ^2^Graduate School of China Academy of Chinese Medical Sciences, Beijing, China; ^3^Institute of Acupuncture and Moxibustion, China Academy of Chinese Medical Sciences, Beijing, China; ^4^Dongzhimen Hospital, Beijing University of Chinese Medicine, Beijing, China; ^5^Affiliated Hospital of Traditional Chinese Medicine, Southwest Medical University, Luzhou, China

**Keywords:** treatment-resistant depression, transcutaneous auricular vagus nerve stimulation, rest-state functional magnetic resonance imaging (rs-fMRI), amplitude of low-frequency fluctuations, regional homogeneity, functional connectivity, orbital frontal cortex

## Abstract

**Objective:**

Transcutaneous auricular vagus nerve stimulation (taVNS) is effective for treatment-resistant depression (TRD). In the current study, we observed the immediate modulating brain effect of taVNS in patients with TRD using rest-state functional magnetic resonance imaging (rs-fMRI).

**Method:**

Forty patients with TRD and forty healthy controls (HCs) were recruited. Rs-fMRI was performed before and after 30 min of taVNS at baseline. The brain regions that presented significantly different the Regional Homogeneity (ReHo) between the TRD patients and HCs were selected as the ROI to calculate the functional connectivity (FC) of full brain. The correlations were estimated between the clinical scales' score and the functional brain changes.

**Results:**

Following taVNS stimulation treatment, TRD patients showed significantly reduced ReHo in the medial orbital frontal cortex (mOFC) (*F* = *18.06, P* < 0.0001), ANCOVA of the mOFC-Based FC images revealed a significant interaction effect on the left inferior parietal gyrus (IPG) and left superior marginal gyrus (SMG) (*F* = *11.6615, P*<*0.001,F* = *16.7520, P*<*0.0001*). Among these regions, the HAMD and HAMA scores and ReHo/FC changes were not correlated.

**Conclusion:**

This study applied rs-fMRI technology to examine the effect of taVNS stimulation treatment on the brain activity of TRD. These results suggest that the brain response of TRD patients to taVNS treatment may be associated with the functional modulation of cortical regions including the medial orbital frontal cortex, the left inferior parietal gyrus, and the left superior marginal regions. Changes in these neuroimaging indices may represent the neural mechanisms underlying taVNS Immediate Stimulation treatment in TRD.

## Introduction

Major depressive disorder (MDD) is a common clinical disorder of the psychiatric system, characterized by persistent depressed mood, reduced interest and cognitive function, anhedonia, and somatic disturbances (1). MDD contributes significantly to the global disease burden, with up to one-third being treatment-resistant patients (2). In clinical treatment, about 35% of patients with depression exhibit poor efficacy even after a complete course of treatment with two or more antidepressants that possess different chemical structures; this type of depression is categorized as treatment-resistant depression (TRD) (3). In addition, the disability and fatality rates of TRD patients are significantly higher than those of ordinary depression patients (4). Serretti et al. (5) reported six most likely risk factors for TRD, including long course of disease, slow onset, comorbid anxiety, advanced age, episode severity, and depressive characteristics. Therefore, TRD is a hot but difficult research topic for psychiatrists at present.

TRD is a complex disorder for which the pathogenesis is not fully understood. Studies have demonstrated TRD is associated with functional abnormalities in brain neural circuits related to emotional and cognition processing, self-representation, and reward processing, these brain regions include the medial orbital frontal cortex (mOFC), amygdala, inferior parietal gyrus (IPG), and superior marginal gyrus (SMG) (6–9). It was reported that anhedonia is associated with neurological dysfunctions in the reward system (10). Additional studies (11–13) revealed the reward loop nervous system carries emotional or cognitive information and decision-making information in the prefrontal cortex. The mOFC is a key part that mediates pain experience and motivation to avoid pain.

TRD treatment is mainly based on drug therapy combined with non-drug treatment. Most antidepressants cause adverse reactions, such as cardiovascular disease and metabolic syndrome (14). Non-drug treatments mainly include psychotherapy, electroconvulsive therapy (ECT), repetitive transcranial magnetic stimulation (rTMS), deep brain stimulation (DBS), and vagus nerve stimulation (VNS). VNS is an FDA-approved somatic treatment for treatment-resistant depression (TRD) that can produce clinically significant antidepressant effects (15). However, the application is limited by the involvement of surgery and potential side effects. To overcome the potential barriers to applying VNS, a non-invasive transcutaneous vagus nerve stimulation (taVNS) method has been developed. The rationale for using taVNS on the ear is based on anatomical studies that suggest the ear is the only place on the surface of the human body where there is afferent vagus nerve distribution (16, 17). Thus, direct stimulation of the afferent nerve fibers on the ear should produce an effect similar to classic VNS in reducing depressive symptoms but without the burden of surgical intervention (18, 19). Our previous research group (20–22) discovered that taVNS is clinically effective in treating TRD and further observed that taVNS has a significant synergistic effect on TRD patients in maintaining drug treatment. The taVNS therapeutic mechanism may be related to the modulating brain default mode network (DMN), reward network and salience network. However, the mechanism of the immediate effect of taVNS in the treatment of TRD remains unclear.

Resting-state functional magnetic resonance imaging (rs-fMRI) is a neuroimaging technique based on blood oxygenation level dependent (BOLD) levels to detect brain activity patterns and is one of the main methods to study the brain effects of acupuncture (23). Additionally, rs-fMRI has been gradually applied in the field of bipolar disorder (24), schizophrenia (25), autism (26), and other psychiatric disorders. Also, rs-fMRI has been applied to study the subtypes of depression (27–29). ReHo is used to assess the level of coordination of neural activity in local brain regions by calculating ReHo values, which indirectly reflect the spontaneous activity of local neurons in time synchronization (30). Functional connectivity (FC), which is a fMRI method for observing the functional association between different brain regions by analyzing the statistical correlation between the time series of different brain regions (31), has also been used in major depressive disorder research.

## Materials and methods

### Recruitment of participants

Forty adult patients aged between 18 and 70 years with a Diagnostic and Statistical Manual of Mental Disorders-IV-Text Revision or 5 (DSM-IV-TR or 5) diagnosis of major depressive disorder who had failed to respond to at least two different antidepressants with adequate dosage and treatment duration (i.e., fluoxetine ≥ 20 mg/day for ≥ 60 consecutive days) were included in our study. Forty healthy controls (HCs) were recruited and matched with patients in sex, age, and education. The HCs had no lifetime history of major or minor psychiatric disorders. In addition, the TRD patients and HCs did not have major medical or neurological illnesses, or a history of alcohol or substance abuse. All participants were right-handed. Before the study, they were all informed of the study protocol and volunteered to participate in the study. Participants with fMRI contraindications and severe organic or mental diseases were excluded.

### Ethical review and registration

The present study was reviewed and approved by the Ethics Committee of Guang'anmen Hospital, China Academy of Chinese Medical sciences (No. 2017-021-SQ) and registered at the Chinese Clinical Trial Registry (No. ChiCTR-1800014277).

### Transcutaneous auricular vagus nerve stimulation

**Figure 1:**
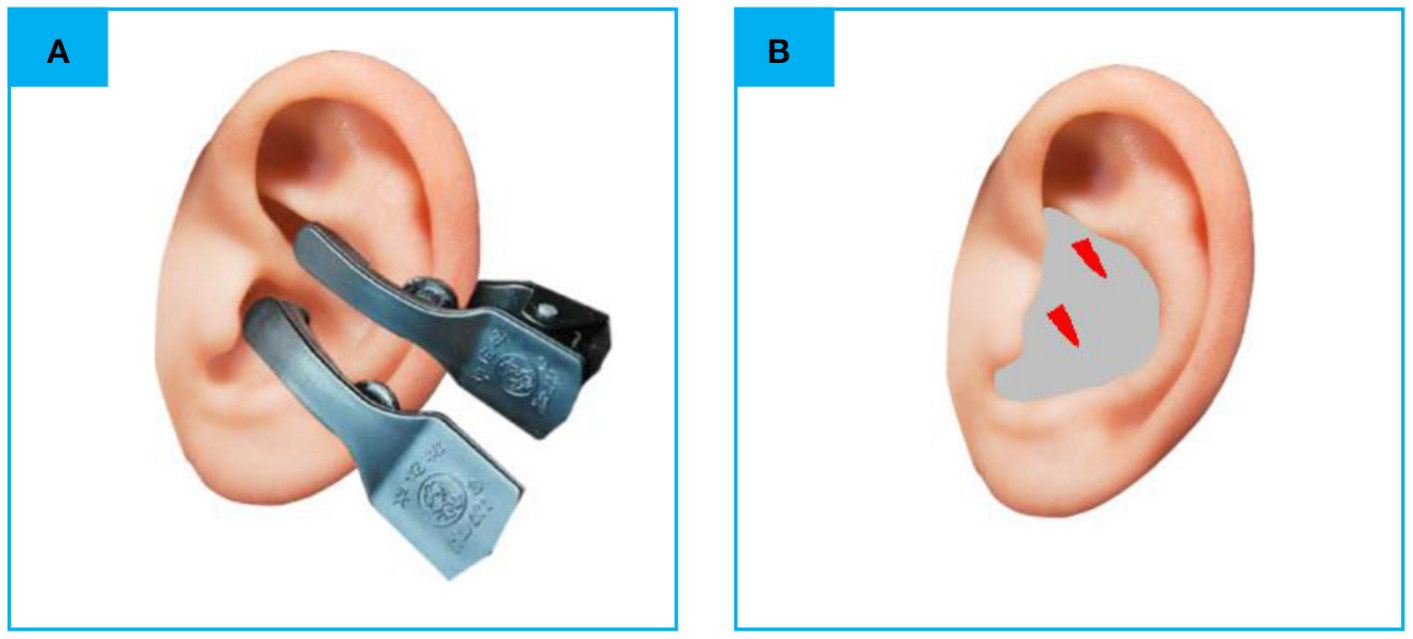
**(A)** The electrodes were attached to the surface of cymba conchae. **(B)** The stimulating place of taVNS. rs-fMRI, rest-state functional Magnetic Resonance Imaging; taVNS, Transcutaneous Auricular Vagus Nerve Stimulation (the red color areas).

The electro-acupuncture stimulator (SDZ-IIB, Hwato brand, manufactured in Su Zhou, China) was attached to the bilateral cymba conchae through electrodes on the skin surface (Figure 1). Parameters were set according to previous studies of taVNS (32, 33): Dilatational wave of 4/20 Hz and pulse width of 0.2 ms ± 30%. The current intensity was adjusted according to each patient s subjective feeling. Each taVNS session lasted for 30 min. Before treatment, the patient's ear armor was routinely disinfected with 75% alcohol.

### Clinical assessments

**Figure 2:**
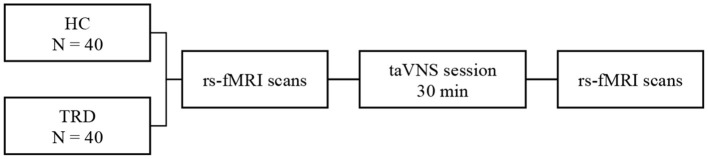
fMRI for instant taVNS were measured before and after the first treatment on the two groups.

All participants accepted Hamilton Rating Scale for Depression (HAMD) and Hamilton Anxiety Rating Scale (HAMA) to estimate the mental status of all the participants. Inclusion in the current study required patients to score >17, and the HCs would be excluded with a total score of HAMD or HAMA >7. The process of the current study is shown in Figure 2. In addition, we screened all patients' T2-weighted and structural images to rule out most of the severe metabolic or immune-related neuropsychiatric diseases, cerebrovascular diseases, inflammatory diseases of the central never system, and intracranial tumors.

### Scan acquisition

Rest-state functional magnetic resonance imaging (rs-fMRI) was performed before and after the first 30 min taVNS session. Participants were told to keep their eyes closed and not fall asleep during the scan. The fMRI data were acquired by Siemens 3.0T Skyra scanner (Siemens; Munich, Germany). The scanning parameters were as follows. The BOLD gradient Echo Planar Imaging (EPI) sequence was used in functional images. Two hundred volumes lasted 6 min and 10 s, repeat time/echo time: 2,000/30 ms, flip angle = 90°, scanning field of view: 224 mm × 224 mm, matrix: 64 × 64, number of layers: 32, layer thickness/spacing: 3.5/0.6 mm. In a high-definition structure image, three-dimensional magnetization was used to prepare a fast gradient-echo sequence, repeat time/echo time: 2,530/2.98 ms, flip angle: 7 degrees, field of view: 256 mm × 256 mm, matrix: 64 × 64, and Layer thickness/spacing: 1.0/0 mm. We obtained 192 images.

### Image processing

#### fMRI data pre-processing

DPABI (http://rfmri.org/DPABI) software (34), an SPM-based functional MRI pre-processing pipeline, was used for data pre-processing. The pre-processing steps were as follows. DICOM file was converted into NIFTI, and the first 10 time points were removed. The remaining 190 time points were slice-time corrected and realigned according to the Friston 24-parameter model. The nuisance signals, including linear trend, head-motion, signals of cerebrospinal fluid, and white matter, were regressed from the data (35). Then, the functional images were co-registered to the T1-weighted structural images, segmented through Voxel-Based Morphometry (VBM). Derived images were normalized to Montreal Neurological Institute (MNI) space according to transformation parameters estimated by VBM. All data used in this study satisfied the criteria of spatial movement in any direction <1.5 mm or degree. Subjects demonstrated no significant group differences in head-motion parameters. In this study, we failed to find significant differences in FD between groups (*F* = *0.4939, P* = *0.4843*).

The limitations of the signal-to-noise ratio and disputes in sampling and pre-processing strategies for fMRI data in the existing voxel-based analysis studies are sometimes contradictory. To better present the short-time intervention, we employed ReHo and FC to reveal the reproductive results.

Regional homogeneity (ReHo) is calculated using voxel-based Kendall's coefficient of concordance (KCC) for the time series of a given voxel with its nearest neighbors (24). ReHo maps were calculated using the unsmoothed and filtered (0.01–0.08Hz) images to remove physiological signals, such as heartbeat and respiration. Then ReHo maps were taken to mean ReHo maps by subtracting the mean voxel-wise ReHo in the entire brain and standardized into Z-value (zReHo Maps). Calculated zReHo maps were smoothed to MNI space with 6 mm Gaussian kernel full width at the half maximum smooth nucleus.

FC is the Pearson's correlations of the temporal fMRI signals between a Region of Interest (ROI) and all brain. Positive brain regions after ReHo statistics found by the above voxel based analyses would be used as the ROI for seed to voxel FC analysis. The AAL template of the WFU_Pick Atlas_v3.0 software was used to extract the seed points of the differential brain regions (36), Calculate the correlation coefficient (r) between the average time series of different brain regions and the time series of other voxels in the whole brain, Pearson's correlation coefficients were transformed into normally distributed scores according to the Fisher's R- to -Z transformation. Statistical inferences were the same as in the ReHo analysis.

### Statistics

#### Clinical data analysis

Clinical data were analyzed using the SPSS 23.0 statistical software (IBM Corporation, Somers, New York). One-way analysis of variance (ANOVA) was used to compare age and education level among the groups, and the chi-square test was used to compare sex. A two-sample t-test was used to compare HAMD-17 and HAMA scores between the two groups, with *P* < 0.05 (two-tailed) as the threshold for statistical significance.

#### fMRI data analysis

In SPSS 25 (SPSS Inc., Chicago, IL, USA), two-sample *t*-tests and χ2 tests were applied to compare the baseline characteristics between the TRD and HC groups.

For the fMRI data, to determine the group × stimulation interaction effect between the two groups and the two scans, the main effects of group (the TRD group and the HC group) and time (baseline and post taVNS stimulation period), Covariates in the repeated measures ANCOVA and *post hoc* analyses were performed. Gender, age, education level, and framewise displacement (FD) metric (derived from Jenkinson's formula) of the four groups of subjects were used as covariates. The brain regions showing significant time differences in the HC group were excluded (37). The result for ANCOVA was the performance in Gaussian random field correction (GRF), combined voxel-wise *P*-value < 0.01 with cluster *P*-value < 0.05 (two-tailed). We performed *post-hoc t*-test analysis using DPARSF 5.1 software for two-by-two comparisons between groups, and Bonferroni correction was applied to the results, setting a threshold of *P* < 0.0125(0.05/4) for statistical significance.

To clarify the behavioral associations of brain function, we performed Pearson correlation analyses between the fMRI values and clinical scales in SPSS 25.

## Results

### Demographic characteristics and clinical results

The demographic and behavioral data are provided in Table 1, in which no significant differences in age and sex between TRD patients and HCs were observed. However, the HAMD and HAMA scores were higher for the TRD patients group (*n* = *40:40, P*<*0.01*).

**Table 1:** Demographic and clinical characteristics of the study participants.

**Items**	**TRD** **(*N =* 40)**	**HC** **(*N =* 40)**	***Z*/χ^2^**	***P-*value**
Age (year)	43.01 ± 11.90	38.33 ± 13.04	1.764	0.082
Sex (M/F)	16/24	13/27	0.487	0.32
Education(year)	13.59 ± 3.63	15.07 ± 5.38	−1.489	0.141
HAMD	22.10 ± 4.33	2.40 ± 1.82	−7.688	<*0.01*
HAMA	23.97 ± 8.95	2.85 ± 2.39	−7.512	<*0.01*

Z, Wilcoxon rank testing; χ 2, chi-square testing. TRD, treatment-resistant depression; HC, healthy control. HAMD, Hamilton rating scale for depression; HAMA, Hamilton anxiety rating scale.

### fMRI results

#### ReHo

**Figure 3:**
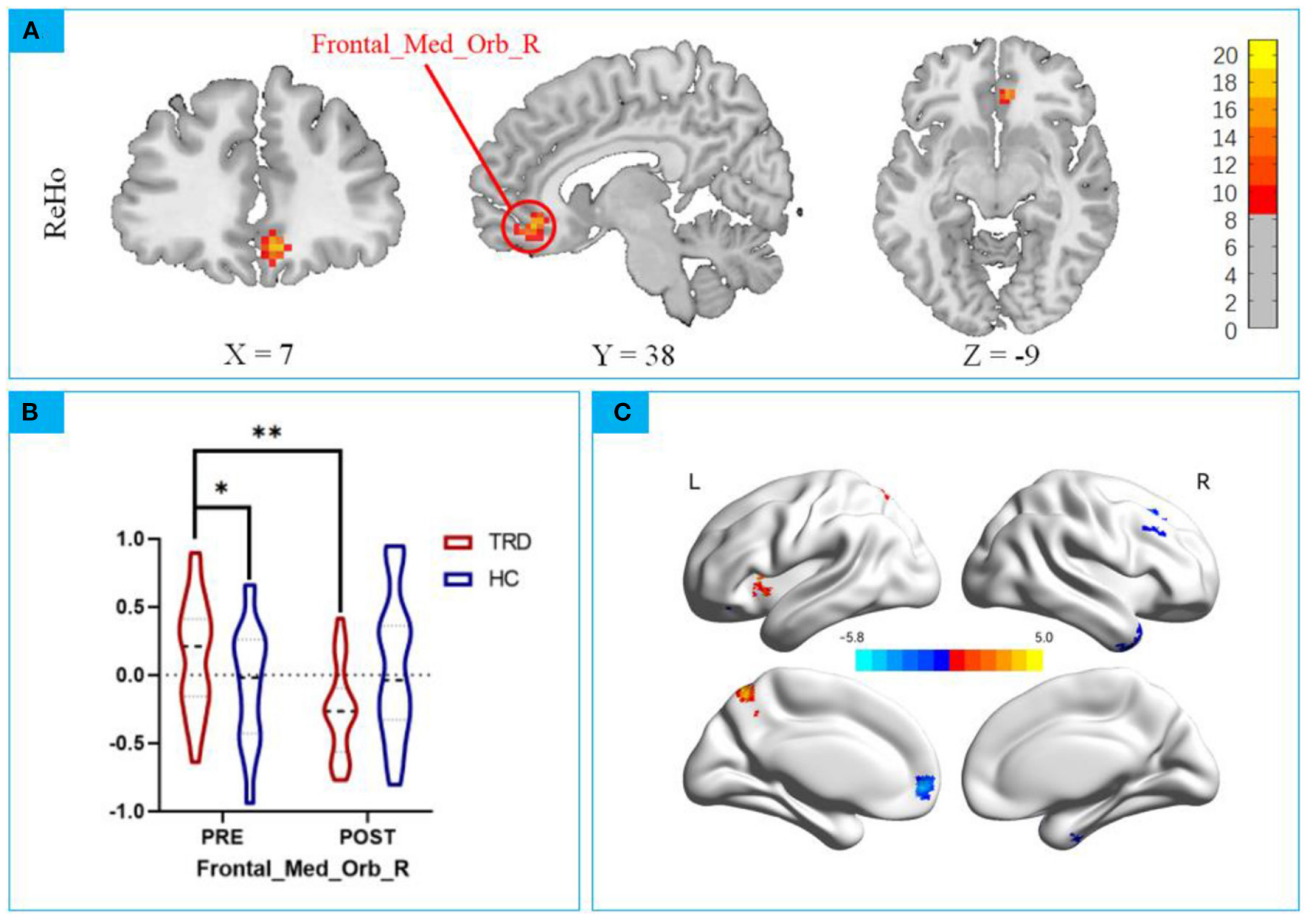
**(A)** Significant group × stimulation interactions on ReHo were observed in the right medial orbital frontal cortex; **(B)** *post hoc* analysis showed taVNS decreased lower activation in the TRD group than baseline. * *p* < *0.01;* ** *p* < *0.001*. **(C)** Main Effect of Time on ReHo. Blue colors represent decreased ReHo in after taVNS stimulation compared to before, while the hot colors represent the opposite.

Group × stimulation interaction differences in ReHo are shown in Figure 3A and Tables 2, 3. Significant group × stimulation interactions on ReHo were observed in the right medial orbital frontal cortex.

**Table 2:** Brain changes with Group × stimulation interaction.

**Items**	**Brain regions (AAL)**	**BA**	**MNI (mm)**			**Number of voxels**	**Peak intensity**
			***X***	***Y***	***Z***		
ReHo	Frontal_Med_Orb_R	11	6	39	−12	42	16.1717

ReHo, Regional homogeneity in the right medial orbital frontal; AAL, Anatomical Automatic Labeling; MNI, Montreal Neurological Institute; BA, Brodmann area.

**Table 3:** Repeated measures ANCOVA of TRD and HC at baseline and post taVNS stimulation period.

**Variables**	***F***	***P***
Time	4.870	0.0303
Group	0.1349	0.7143
Time × Group	18.06	<0.0001

Covariates in the repeated measures ANCOVA include gender, age, education level, and FD.

Repeated measures ANCOVA revealed a significant interaction effect on the right medial orbital frontal cortex (*F* = *18.06*, P < 0.0001, Figure 3A), *post hoc* analyses confirmed that the Reho value in the mOFC of the TRD group was significantly higher in the HC group in the baseline (*t* = *2.402, P* < *0.001;* Figure 3B). After instant taVNS stimulation, the ReHo value was significantly decreased (*t* = −*4.314, P* < *0.001;* Figure 3B), Before and after treatment in the HC group, the difference was not statistically significant (*t* = *1.155, P* = *0.2515;* Figure 3B).

Significant main effect on time was found, Compared to before taVNS stimulation, in the right posterior lobes of the cerebellum, temporal inferior gyrus, left medial orbital frontal, and right superior frontal gyrus of the ReHo value decreased, Left precentral gyrus of the ReHo value increased (Table 4). No significant main effect on group effect was found. The 3D map is produced by the BrainNet Viewer toolbox (38) (Figure 3C).

**Table 4:** Anatomical Locations of Significant Main Effect of Group on ReHo.

**Items**	**Brain regions (AAL)**	**BA**	**MNI (mm)**			**Number of voxels**	**Peak intensity**
			***X***	***Y***	***Z***		
ReHo	Cerebelum_Crus2_R	–	33	−81	−48	205	−5.8082
ReHo	Temporal_Inf_R	20	42	−6	−39	168	−3.8751
ReHo	Frontal_Med_Orb_L	11	−12	54	−3	160	−4.9271
ReHo	Frontal_Sup_R	9	24	15	39	141	−4.5308
ReHo	Precuneus_L	7	−9	−63	60	128	4.8316

#### FC

**Figure 4:**
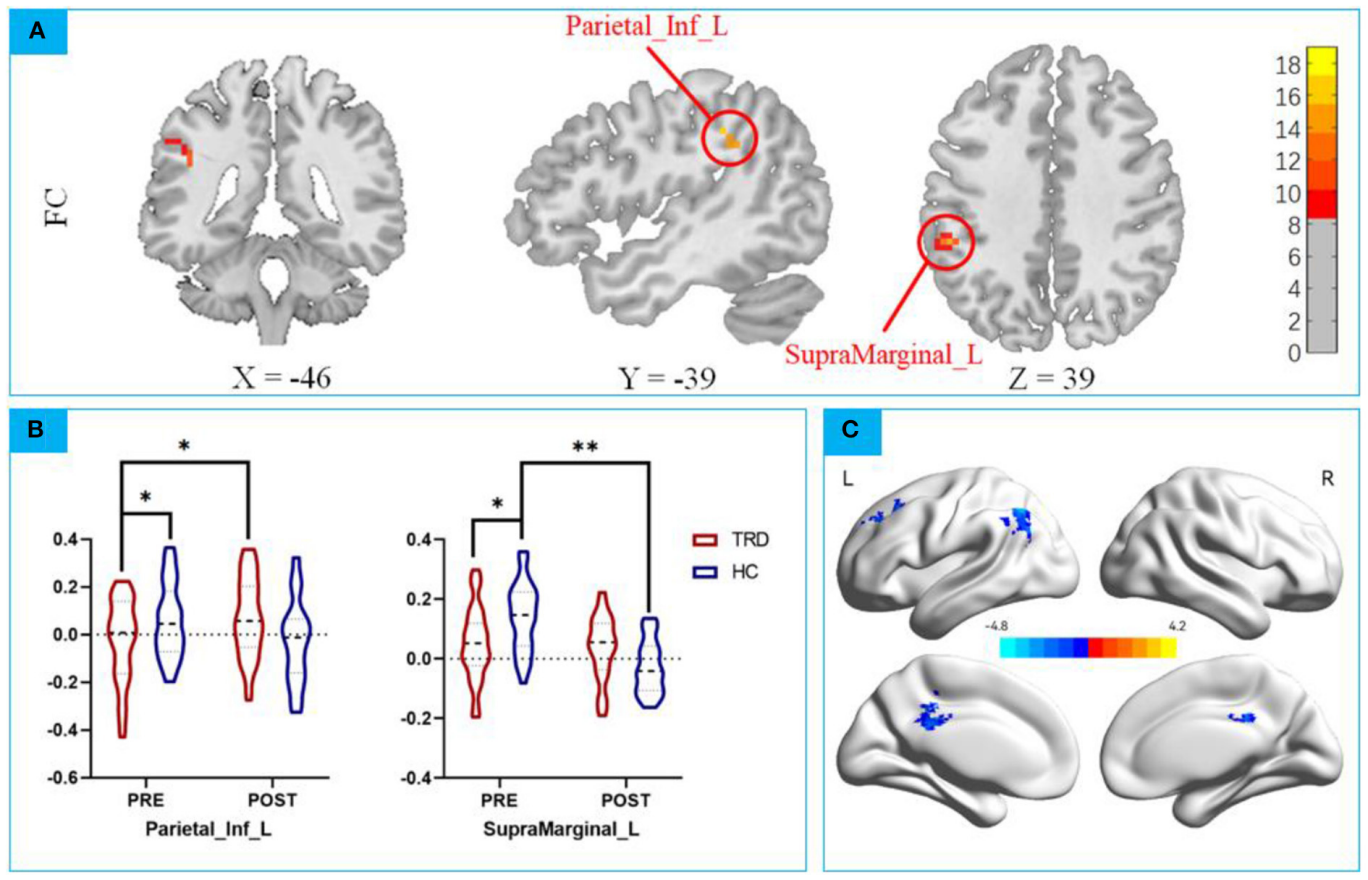
**(A)** Repeated measures ANCOVA revealed a significant interaction effect on the left inferior parietal gyrus and left superior marginal gyrus; **(B)** *post hoc* analysis mOFC-Based FC between the mOFC and IPG; *post hoc* analysis mOFC-Based FC between the mOFC and SMG. * *p* < 0.01; ** *p* < 0.001; **(C)** Main Effect of Time on FC of mOFC and SMG. Blue colors represent decreased FC of mOFC and SMG in POST-taVNS stimulation compared to PRE-taVNS.

According to the ReHo results, we defined the right medial orbital frontal regions as ROI for the FC analyses (39). Repeated measures ANCOVA revealed a significant interaction effect on the left inferior parietal gyrus and left superior marginal gyrus (*F* = *11.6615, P* < *0.001, F* = *16.7520, P* < *0.0001;* Figure 4A; Tables 5, 6).

**Table 5:** Group × Time Interaction on the mOFC-Based FC.

**Items**	**Brain regions (AAL)**	**BA**	**MNI (mm)**			**Number of voxels**	**Peak intensity**
			***X***	***Y***	***Z***		
FC	Parietal_Inf_L	40	−54	−40	39	20	15.466
FC	SupraMarginal_L	48	−48	−40	33	20	15.466

FC, Functional connection in the left inferior parietal gyrus and left superior marginal gyrus; AAL, Anatomical Automatic Labeling; MNI, Montreal Neurological Institute; BA, Brodmann area.

**Table 6:** Repeated measures ANCOVA of TRD and HC at baseline and post taVNS stimulation period.

**Variables**	**Parietal_Inf_L**		**SupraMarginal_L**	
	***F***	***P***	***F***	***P***
Time	0.0193	0.8898	4.4858	0.0374
Group	0.1269	0.7226	0.2614	0.6106
Time × Group	11.6615	*0.0010*	16.7520	*0.0001*

ANCOVA of the mOFC-Based FC images showed that the group × time interaction effect of the mOFC with IPG showed statistical significance. *Post hoc* analyses confirmed that the FC strength in the TRD group was significantly lower in the HC group in the baseline (*t* = *2.133, P* < *0.001;* Figure 4B), after instant taVNS stimulation, the FC strength was significantly increased *(t* = −*4.314, P* < *0.001;* Figure 4B). Before and after treatment in the HC group, the difference was not statistically significant (*t* = *1.155, P* = *0.2515;* Figure 4B).

ANCOVA of the mOFC-Based FC showed that the group × time interaction effect of the mOFC with the SMG showed statistical significance. *Post hoc* analyses confirmed that the FC strength in the TRD group was significantly lower in the HC group in the baseline (*t* = *3.236, P* < *0.01;* Figure 4B), after instant taVNS stimulation, the FC strength was increased (*t* = *1.623, P* = *0.11339*; Figure 4B), but the difference was not statistically significant. Furthermore, after treatment in the HC group, the FC strength was significantly lower in the baseline (*t* = 8.704, *P* < 0.001; Figure 4B).

No significant main effect was found in functional connection of mOFC and IPG. However Significant main effect on time was found in mOFC and SMG (Table 7), Compare before taVNS stimulation in the left middle Cingulate Gyrus, left middle frontal gyrus, and left Inferior parietal of the FC strength decreased. No significant main effect on group effect was found. The 3D map is produced by the BrainNet Viewer toolbox (38) (Figure 4C).

**Table 7:** Anatomical Locations of Significant Main Effect of Group on mOFC-based FC of mOFC and SMG.

**Items**	**Brain regions (AAL)**	**BA**	**MNI (mm)**			**Number of voxels**	**Peak intensity**
			***X***	***Y***	***Z***		
FC	Cingulum_Mid_L	23	−6	−39	39	159	−4.3617
FC	Parietal_Inf_L	40	−45	−60	48	165	−4.7134
FC	Frontal_Mid_L	9	−24	33	39	132	−3.9720

### Correlation analyses

**Figure 5:**
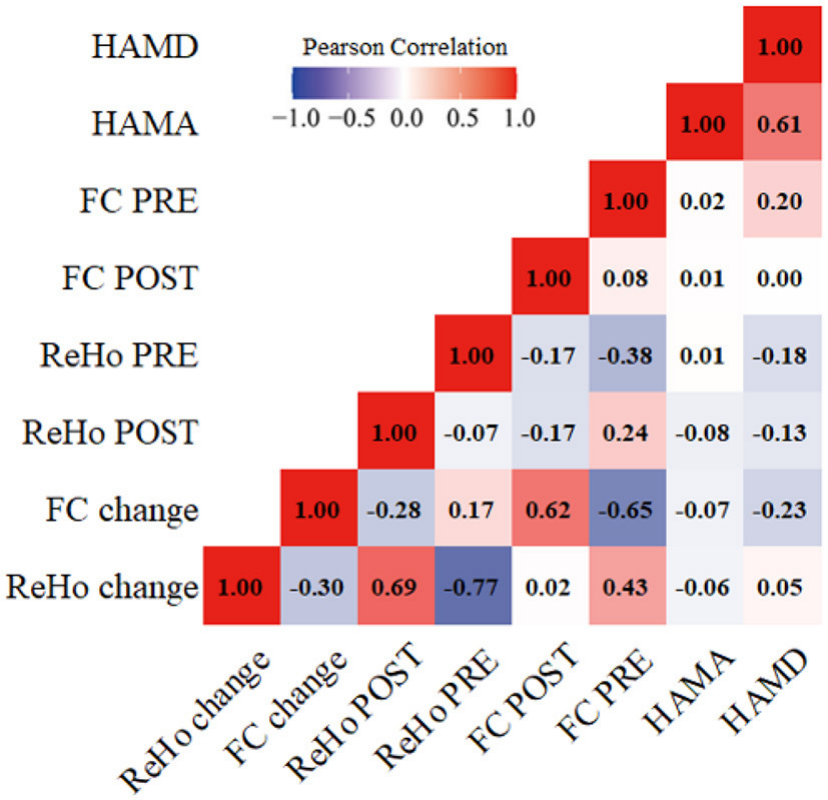
Correlations between the clinical scales' scores and the fMRI values. HAMD, Hamilton rating scale for depression; HAMA, Hamilton anxiety rating scale. ReHo, Regional homogeneity; FC, Functional connectivity; ReHo PRE/FC PRE, before taVNS treatment; ReHo POST/FC POST, after taVNS treatment; ReHo change/FC change, the difference in value before and after treatment.

The HAMD and HAMA scores and ReHo/FC changes in the above-mentioned brain regions were not correlated (Figure 5).

## Discussion

This study applied rs-fMRI technology to examine the effect of taVNS stimulation treatment on the brain activity of TRD. Our current study revealed that following taVNS stimulation treatment, TRD patients showed significantly reduced ReHo in the medial orbital frontal cortex (mOFC). ANCOVA of the mOFC-Based FC images revealed a significant interaction effect on the left inferior parietal gyrus (IPG) and left superior marginal gyrus (SMG). Among these regions, the HAMD and HAMA scores and ReHo/FC changes were not correlated.

### taVNS can immediately regulate the synchrony of neuronal activity in the mOFC brain region of TRD patients

Several studies have confirmed that patients with TRD tend to have lower reward sensitivity (40). The mOFC is an integral part of the reward network and is associated with emotional information and sensory stimuli (41, 42). Fang et al. (43) illustrated that abnormal OFC-default network functional connection regulation was significantly related to relieving depressive symptoms. Studies have also demonstrated that the gray matter volume of OFC and the functional connection of OFC-amygdala in TRD patients are positively correlated, reflecting that TRD patients may suffer from greater stress and depression, and must call OFC more frequently to regulate the amygdala response to negative emotions (44). Compared with the HC group, it was found that ReHo in the mOFC brain region of TRD patients was decreased by taVNS immediate treatment. Based on previous studies (20–22), taVNS may have an immediate regulation effect on the spontaneous brain activity of mOFC in TRD patients to improve the status of the limbic system and reward circuit. The mOFC is also a key brain region involved in safety and risk decision-making. When TRD shows overactivation of OFC in the resting state, it will overreact to social rejection signals, thus increasing the risk of suicidal behavior. In addition, from the perspective of neural circuits, mOFC is also involved in the motivation control of punishment avoidance conditions, suggesting that its significant activation may simultaneously mediate the process of individuals' high avoidance motivation for pain (45). More than half of TRD patients report suicidal thoughts (46). After taVNS treatment, TRD patients' ReHo value immediately decreased, and the synchronization of neuronal activity was significantly reduced, which means that taVNS can effectively inhibit negative emotions such as suicidal tendencies in TRD patients. In conclusion, taVNS treatment may activate the emotion cognitive regulation function involved in mOFC and jointly regulate the negative emotions of TRD patients.

### taVNS has an immediate regulatory effect on brain regions and brain networks related to the regulation of emotion

In this study, ANCOVA of the mOFC-Based FC images revealed a significant interaction effect on the left inferior parietal gyrus (IPG) and left superior marginal gyrus (SMG). Furthermore, Main Effect of Time on ReHo, Compared before taVNS stimulation, in the right posterior lobes of the cerebellum, temporal inferior gyrus, left medial orbital frontal and right superior frontal gyrus of the ReHo value decreased, left precentral gyrus of the ReHo value increased. Main Effect of Time on FC of mOFC and SMG, compare before taVNS stimulation, in the left middle Cingulate Gyrus, left middle frontal gyrus, and left Inferior parietal of the FC strength decreased. Abnormalities in these regions have also been extensively reported in previous studies, and the present study has accumulated more evidence for the relevant results (47–49). Previous studies have posited that rumination may play a pivotal role in the psychopathology of TRD (50, 51), Default mode network (DMN), such as the medial prefrontal cortex (MPFC) and posterior cingulate cortex/precuneus. Frontoparietal control network (FPCN) regions, including the inferior parietal lobule (IPL), dorsal lateral prefrontal cortex (DLPFC), and superior marginal gyrus (SMG) (52). DMN and FPCN are closely related to emotion and cognitive processing (53), Silani et al. (54) showed that the SMG is a key brain area for emotion control. The FPCN anatomically connects the DMN and the dorsal attention network (DAN), and its function is to integrate stored internal representations with external environmental information, and to simultaneously resolve multiple interdependent emergencies and response mappings to conflicting stimuli, assigning work. Memory and attention resources. Our study found that the FC strength in the TRD group was significantly lower in the HC group in the baseline that the top-down regulation of TRD emotion is abnormal. And taVNS treatment can reduce the neural activity level of the mOFC and increase the neural activity intensity of the SMG and the IPL, which has a dynamic regulatory effect on the brain function of TRD patients. This suggests that taVNS can regulate the negative emotions of TRD from bottom to top (55). In conclusion, taVNS has an immediate regulatory effect on brain regions and brain networks related to the regulation of emotion.

Interestingly, we also found that after taVNS intervention in the HC group, the FC strength was significantly lower in the baseline. The taVNS immediate stimulation also had modulating effects in healthy individuals. Previous studies have also found short-term antidepressant therapy for healthy individuals reduce activity in the amygdala, OFC, superior frontal gyrus, and precentral gyrus, and SMG during emotional stimulation. These brain regions are associated with the negative affective of depression (56–64). Our taVNS treatment is consistent with studies on the regulatory effect of antidepressants on healthy individuals, which is worthy of further study.

### Limitations

First, the study's sample size is small, and thus the results may be biased. Fewer brain areas are immediately adjusted to cause changes, which may be different from those after long-term treatment. Second, this study only used the commonly used research indicators of resting-state fMRI to observe the changes in the immediate effect mechanism of the brain, and the indicators used are not comprehensive enough.

In our future studies, the sample size will be expanded, and a variety of functional imaging research methods will be used to further explore the brain mechanism of the efficacy of taVNS on TRD patients. More indicators, including arterial spin labeling (ASL), GABA and other indicators of TRD patients, will need to be carried out for statistical analysis to improve the scientific value of this study.

## Conclusions

In this study, we found taVNS can immediately regulate the synchrony of neuronal activity in the mOFC brain region of TRD patients. ANCOVA of the mOFC-Based FC images revealed a significant interaction effect on the IPG and SMG. In summary, the potential mechanism of taVNS treatment for TRD may be to enhance the function of emotion regulation circuits, monitor and manage negative emotions. Activity of emotion-processing networks, reduces the processing of negative emotions in TRD. Through taVNS treatment, the abnormal brain regions in TRD can be normalized, or even reversed, which may play a compensatory role in the reduction of depressive symptoms and involving DMN, FPCN and Reward Network.

## Data availability statement

The original contributions presented in the study are included in the article/supplementary material, further inquiries can be directed to the corresponding authors.

## Ethics statement

The studies involving human participants were reviewed and approved by Ethics Committee of Guang'anmen Hospital, China Academy of Chinese Medical Sciences, China (No. 2017-021-SQ). The patients/participants provided their written informed consent to participate in this study. Written informed consent was obtained from the individual(s) for the publication of any potentially identifiable images or data included in this article.

## Author contributions

JF conceived and designed this experiment. This article was written mainly by YM and ZW. Patients were recruited and assessed by JS and CG. fMRI data were collected by ZW and JH. YM and ZD drew the diagrams and carried out the statistical analysis of data. YH and LZ performed fMRI on the subjects. JF and YLi reviewed the article. Text correction was done by YLu and LC. All authors contributed to the article and approved the submitted version.

## Funding

This research was supported by the Science and Technology Innovation Project of China Academy of Chinese Medical Sciences (CI2021A03301), National Natural Science Foundation of China (82174282 and 81774433), and National Key Research and Development Program of China (2018YFC1705800).

## Conflict of interest

The authors declare that the research was conducted in the absence of any commercial or financial relationships that could be construed as a potential conflict of interest.

## Publisher's note

All claims expressed in this article are solely those of the authors and do not necessarily represent those of their affiliated organizations, or those of the publisher, the editors and the reviewers. Any product that may be evaluated in this article, or claim that may be made by its manufacturer, is not guaranteed or endorsed by the publisher.
